# The XBabelPhish MAGE-ML and XML Translator

**DOI:** 10.1186/1471-2105-9-28

**Published:** 2008-01-18

**Authors:** Don Maier, Farrell Wymore, Gavin Sherlock, Catherine A Ball

**Affiliations:** 1 Department of Biochemistry, Stanford University School of Medicine, Stanford, CA 94305-5307, USA; 2 Department of Genetics, Stanford University School of Medicine, Stanford, CA 94305-5120, USA

## Abstract

**Background:**

MAGE-ML has been promoted as a standard format for describing microarray experiments and the data they produce. Two characteristics of the MAGE-ML format compromise its use as a universal standard: First, MAGE-ML files are exceptionally large – too large to be easily read by most people, and often too large to be read by most software programs. Second, the MAGE-ML standard permits many ways of representing the same information. As a result, different producers of MAGE-ML create different documents describing the same experiment and its data. Recognizing all the variants is an unwieldy software engineering task, resulting in software packages that can read and process MAGE-ML from some, but not all producers. This Tower of MAGE-ML Babel bars the unencumbered exchange of microarray experiment descriptions couched in MAGE-ML.

**Results:**

We have developed XBabelPhish – an XQuery-based technology for translating one MAGE-ML variant into another. XBabelPhish's use is not restricted to translating MAGE-ML documents. It can transform XML files independent of their DTD, XML schema, or semantic content. Moreover, it is designed to work on very large (> 200 Mb.) files, which are common in the world of MAGE-ML.

**Conclusion:**

XBabelPhish provides a way to inter-translate MAGE-ML variants for improved interchange of microarray experiment information. More generally, it can be used to transform most XML files, including very large ones that exceed the capacity of most XML tools.

## 1 Background

Researchers using DNA microarrays in their studies are widely encouraged, and sometimes compelled by journals or funding agencies, to increase the usefulness of their individual research efforts by making their experimental information easily accessible to the community at large. Conversely, many researchers would like to gain easy access to microarray data from other experimenters' work. The need to exchange and aggregate large-scale biomedical data motivates requirements for universal and easy-to-use tools and file formats to do this. This, in turn, has engendered efforts to describe microarray experiments in a standard way.

The need for a standard format led to the development of the Microarray Gene Expression Markup Language (MAGE-ML) [1], a standardized Extensible Markup Language (XML) format for describing microarray experiments and their result data. Unfortunately, some characteristics of actual documents have limited MAGE-ML's usefulness as a standard. Specifically:

 MAGE-ML documents can be so large that most native XML viewing and transformation tools are unable to open them. Also, even if MAGE-ML documents were typically of a more modest size that allowed viewing and editing tools to digest them, most working experimental biologists find the XML format challenging to "parse" visually and are unfamiliar with those XML tools that try to ameliorate this problem with a reformatted display.

 The semantics of MAGE-ML are so complex that any two MAGE-ML producers are likely to interpret a given MAGE-ML document differently. Conversely, because there are frequently different choices for representing the same information, different producers are likely to produce different MAGE-ML representations of it. In our discussion, we use the term *semantic variant *to refer to one particular semantic use or interpretation of MAGE-ML that one particular producer generates.

The result of the large file sizes together with the need to deal with many semantic variants is that microarray data are represented in many, sometimes non-interchangeable MAGE-ML documents, with no existing XML approach to reconcile this "Tower of Babel".

Various approaches to transforming MAGE-ML semantic variants are possible. For example, one might import MAGE-ML into a relational (SQL) database, which can handle the large size of the information set. Once in an SQL database, SQL queries can be used to transform the now-relationally represented MAGE-ML. Finally, the results of this SQL transformation can be exported. Unfortunately, this approach has at least two serious drawbacks. First, the description of a microarray experiment in MAGE-ML is complex, multilayered, hierarchical in some parts, DAG (Directed Acyclic Graph)-like in others, and generally interconnected in ways that do not admit a simple relational representation. As a result, it is practically impossible to verify, or even have high confidence that an algorithm for transforming XML into and out of relational tables (including the transformation of XML data types into and out of SQL data types) preserves the intended semantics. Second, even if correct, the import and export of XML into and out of an SQL database is costly and inefficient.

We describe here a new approach to inter-translating different semantic variants of MAGE-ML that does not suffer from these drawbacks.

XBabelPhish is a powerful, general-purpose, native XML document translator. It accepts a MAGE-ML file (or indeed any XML file) for translation and enters it into a native XML database. It then executes XQuery [2] statements supplied in a translation definition to transform the loaded document into the desired XML format. If the original file was MAGE-ML and the translation definition defines a mapping from MAGE-ML to MAGE-ML, then the result of the translation will be MAGE-ML. But depending on the translation definition, the result could be an XML file in any desired format. Finally, XBabelPhish unloads the translated document from the XML database into the native file system.

XBabelPhish runs on most major computing platforms, including Windows, Linux, BSD UNIX, Mac OS/X and all POSIX-compliant operating systems. It should run on the 64-bit versions of these platforms on which Berkeley DB XML runs (see below), but this has not yet been tested.

**Figure 1:**
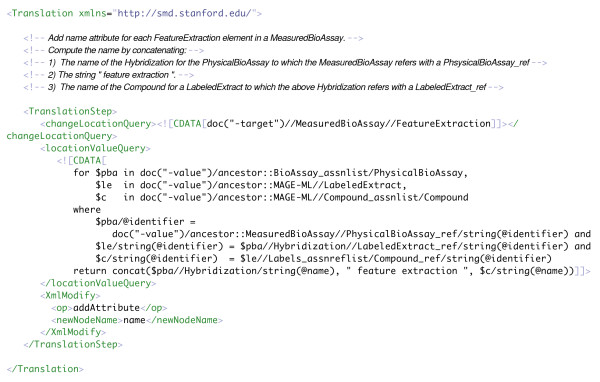
trans_example.xml. A one-step translation that specifies 1) *FeatureExtraction *elements are to be changed (specified by the **changeLocationQuery**), 2) the new content for the change to each selected *FeatureExtraction *(specified by the **locationValueQuery**) is the concatenation of the existing names for a *Hybridization *and a *Compound *related to that *FeatureExtraction *– connected by the string literal " feature extraction ", 3) the change is to add an attribute to each of the *FeatureExtraction *elements (specified as the **op addAttribute**), and 4) the attribute name is "name" (specified as the **newNodeName**).

XBabelPhish exceeds the capabilities of other native XML translation mechanisms, such as XSLT [3] (Extensible Stylesheet Language Transformations, a W3C standard XML-based style sheet language), both in its expressive power and in the size of documents that it can handle. Compared to non-native approaches based on a relational database, it is far simpler to install, manage, and run; and it provides a far simpler and more reliable way to arrive at correct translations. The reliability derives largely from the complete absence of convoluted and impossible-to-verify transformations of the XML into relational tables and back again. Therefore, it is relatively easy to know what a translation does. (See Figure 1 for an example translation definition that is discussed in Section 3.2.)

The simplicity comes from XBabelPhish's ability to define or change a translation with absolutely no programming in the conventional sense. Defining a translation is essentially a matter of writing one or two XQuery statements in a small translation definition document. Changing a translation is simply a matter of changing these XQuery expressions. This also contributes to XBabelPhish's reliability because XBabelPhish does not have to change when the translation does.

The expressive power of XBabelPhish comes from its use of XQuery to define translations. XQuery is a powerful proper superset of XPath [4] (a simple, W3C standard language for navigating the hierarchy of elements and attributes in an XML document) to identify change locations. In contrast, XSLT uses only XPath. XQuery is a W3C standard [5] language that so far lacks an update component. However, XBabelPhish is able to modify XML documents by using Berkeley DB XML's [6] XQuery implementation, which extends standard XQuery with a rich set of modification operations. XBabelPhish's ability to handle large documents comes from its use of Berkeley DB XML as a native XML database. Berkeley DB XML is an open-source product that may be used freely in freely redistributable software such as XBabelPhish. XBabelPhish's platform requirements (above) are just those of Berkeley DB XML.

## 2 Implementation

XBabelPhish is a standalone, pure Java 1.5 program that uses the storage and XQuery services of Berkeley DB XML, a native XML database (written in C++), which in turn, is built on Berkeley DB (written in C).

**Figure 2:**
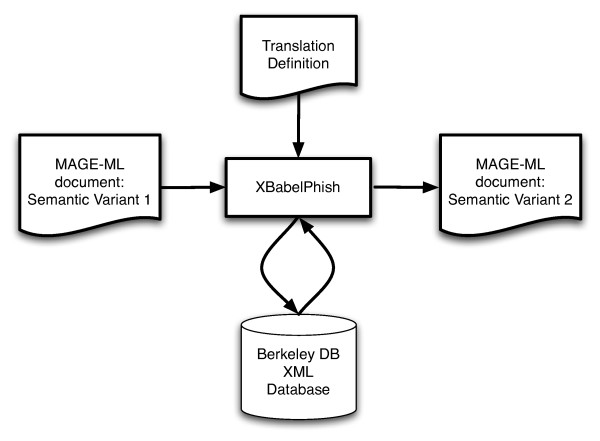
XBabelPhish performing a MAGE-ML document translation. The inputs to XBabelPhish are an XML document (here, one semantic variant of MAGE-ML) to translate, and a translation definition – a typically brief XML description of a sequence of translation steps. XBabelPhish loads both the source document and the translation definition into a Berkeley DB XML database where it performs the XQuery-based updates that implement each of the steps in the translation definition. When the translation is complete, XBabelPhish extracts the translated document from the database to produce the output (here, another semantic variant of MAGE-ML).

XBabelPhish has a very simple *modus operandus*: It streams a user-supplied source XML document into a Berkeley DB XML "container". Once deposited in the XML database, XBabelPhish transforms the source document according to a user-supplied translation definition. Finally, it streams the translated document out of its database container and into a new XML document. This is the translation or required semantic variant of the original source document. This simple arrangement is illustrated in Figure 2.

The translation definition is itself an XML document that XBabelPhish streams into the XML database (see Figure 1 for an example presented in Section 3.2). It is typically quite small, and conforms to a simple XML translation schema defined by XBabelPhish. A translation definition comprises a sequence of translation steps that XBabelPhish executes in a Berkeley DB XML context. Each translation step contains an XQuery statement whose result set is a sequence of XML nodes in the source document. The nodes in this sequence are the locations for possible change. Each step also includes an operation verb that specifies what kind of change to make at each identified change location – for example, insert an element, add an attribute, update an attribute's value, or delete the node. Usually, a translation step also includes a second XQuery statement that computes any new content (the element to be inserted, the attribute to be added, the new value for an update, etc.) for the change.

The second, content-computing XQuery in a translation step may be a "context query" that executes in the context of each change location defined by the first XQuery in the step. In this way, the local XML "environment" around a change location (for example, values of attributes in the immediately containing element) may be used to define the change at that location. Using this device, a single translation step may compute differing, context-appropriate new content for each of any number of change locations. This is a very compact and powerful idiom for specifying how to transform an XML document.

A key attribute of an XBabelPhish translation definition is that it is not tied to any particular XML document. It uses symbolic document references that permit its use in translating a document without regard to name. This facilitates the development of libraries of translations for any XML document domain of interest. In the world of MAGE-ML, work may be done to identify the important semantic variants and develop translations between them. This approach, which would result in n (n - 1) translations for n semantic variants, may be refined into a 2n approach by settling on one canonical variant V_c_, and defining the two translations V_c _→ V_n _and V_n _→ V_c _for each non-canonical variant V_n_.

XBabelPhish works well on very large documents up to and beyond 250 Mb. For documents substantially less than this size and requiring modest numbers of changes, Berkeley DB XML's XQuery engine provides completely adequate performance. For example, in one of our test cases running on a Mac OS X G5 2 GHz processor, XBabelPhish takes around 8 minutes to execute a four-step translation on a 36.5 Mb. MAGE-ML document. For a very large document, or one whose translation involves large numbers of changes, the current version 2.3.10 of Berkeley DB XML has scaling problems that reduce XBabelPhish's translation performance unacceptably. XBabelPhish solves this problem and still works quite well by splitting such a source document into fragments, translating each of the resulting fragments, then reassembling the translated fragments into a single translation result. XBabelPhish uses a streaming, StAX "pull" parser to implement this function efficiently.

Splitting and merging increase translation time by a factor that depends on the number of steps in the translation and the number of fragments into which the source document is split. For the four-step translation of the 36.5 Mb. MAGE-ML document mentioned above, splitting the document into 16 fragments increases the translation time from 8 to 34 minutes. In another of our test cases running on a Mac OS X G5 2 GHz processor, XBabelPhish takes about 140 minutes to execute a two-step translation on a 232.9 Mb. MAGE-ML document by splitting the document into 26 fragments. However, splitting and merging is the only way to translate this larger document; direct translation does not complete within 24 hours. We expect Berkeley DB XML to overcome its scaling problems. This will give XBabelPhish a big performance boost and virtually eliminate the need to split and merge documents.

The XBabelPhish distribution includes a javadoc that completely documents how to install and run the translator. Installation is mostly a matter of installing Berkeley DB XML. Documentation for constructing translation definitions is provided by comments in the translation schema itself. An XBabelPhish command (-printschema) prints this schema. The distribution also provides sample translation definitions and source files drawn from the world of MAGE-ML. These samples illustrate the power of XBabelPhish and provide a guide-by-example for creating translation definitions. For those less familiar with XQuery, there are GUI tools for constructing and validating XQuery statements, such as the <oXygen/> XML editor [7], whose academic license fee is nominal.

It is worth reiterating that absolutely nothing in XBabelPhish ties it to the syntax or semantics of MAGE-ML. It is a completely general XML translator.

## 3 Results

### 3.1 Translation definitions

To have a good understanding of XBabelPhish's capabilities, we need to look at translation definitions. As previously mentioned, an XBabelPhish translation definition takes the form of an XML file that conforms to a translation.xsd built into XBabelPhish. Along with a file to be translated, a translation definition is passed to XBabelPhish to tell it how to perform the translation.

The discussion in the following subsections may be better understood by referring to Figure 1 for the example discussed in Section 3.2.

### 3.1.1 Symbolic names

Translation definitions contain XQuery statements that refer to the document being translated. Typically, there will be many documents with many different names that must be translated in the same way from one semantic variant to another. So that one translation definition can be used for each of these documents, XBabelPhish recognizes symbolic document names in XQuery statements:

 doc("-source"): the unaltered source document being translated.

 doc("-target"): the (possibly) altered document after zero, one, or more translation steps have been executed.

 doc("-value"): an XML value in the result set of a previously executed **changeLocationQuery **(see below) in the same translation step. An XQuery that uses this construct is a "context query".

### 3.1.2 Target locations

As mentioned in Section 2, each step in a translation contains an XQuery statement whose result set is a sequence of (typically element or attribute) nodes that define where that step's changes are to be applied. These are the "change locations" or "targets" for that translation step. The XQuery that defines these targets is either a **changeLocationQuery**, or a **targetWithValueQuery**, as explained in Section 3.1.3.

### 3.1.3 Defining new content

Each operation primitive except **delete **defines new content to apply at each target location. There are three ways to define this new content, listed below. In a translation step that uses either of the first two ways of defining new content, the step's first query is a **changeLocationQuery **whose role is confined to defining change locations. The third way of defining new content is with a **targetWithValueQuery **that defines the change locations *and *the new content in one result set:

1) **newContentQuery**: After a **changeLocationQuery **that identifies the target locations, a **newContentQuery **is executed exactly once in the step; it defines a single, unchanging value that is applied at each target location.

2) **locationValueQuery**: Also used after a **changeLocationQuery**, a **locationValueQuery **is a "context query" (see above). It is executed once for *each *change location to define location-dependent new content.

3) **targetWithValueQuery**: This is the one and only XQuery in the step. It defines *both *the change locations *and *their new content. New content elements in the result set alternate with change location nodes, with the new content for a change location immediately following it. A **targetWithValueQuery **typically uses a FLWOR expression [8]. (A FLWOR expression – For-Let-Where-Order by-Return – is the most common and powerful XQuery construct, similar to SQL's SELECT-FROM-WHERE). In XBabelPhish, it would have the form:

for $x, at $y in doc("-target")//... return (f($x), g($y))

where f() and g() represent some XQuery function. doc("-target") is a symbolic reference to the document being translated – in the transformed state produced by the execution of all preceding translation steps.

See Figure 1 for an example (discussed in Section 3.2) of a translation definition used in a translation.

#### 3.1.4 Translation operations

XBabelPhish provides a rich set of operation (**op**) primitives for adding, deleting, updating, and renaming XML nodes in a document. These primitives focus on the element, attribute, and text nodes that are the ones most commonly requiring transformation.

The primitive operations are presented in Table 1.

**Table 1:** XBabelPhish primitive operations

***Operation***	***Definition***	***Target Location***	***New Content***	***New Content Type***	***Result***
**addElementFirst**	add an element as the first child of a targeted parent element	<x>	<a/>	element to add	<x><a/></x>
**addElementLast**	add an element as the last child of a targeted parent	<x>	<d/>	element to add	<x> <a/> <d/> </x>
**insertElementAfter**	insert an element after a targeted sibling element	<a>	<b/>	element to add	<x> <a/> <b/> <d/> </x>
**insertElementBefore**	insert an element before a targeted sibling element	<d>	<c/>	element to add	<x> <a/> <b/> <c/> <d/> </x>
**addAttribute**	add an attribute to a targeted element	<x>	name	name of attribute to add	<x> name="xFamily" <a/> <b/> <c/> <d/> </x>
			"xFamily"	attribute value	
**addTextStart**	add text to the start of an element	<x>	"start"	text to add	<x> start name="xFamily" <a/> <b/> <c/> <d/> </x>
**addTextEnd**	add text to the end of an element	<x>	"end"	text to add	<x> start name="xFamily" <a/> <b/> <c/> <d/> end </x>
**delete**	delete a target node of any type	<d>		node to delete	<x> start name="xFamily" <a/> <b/> <c/> end </x>
**rename**	rename a target element or attribute node	<a>/@name	title	new name	<x> start title="xFamily" <a/> <b/> <c/> end </x>
**update**	update the value of a target element or attribute node	<a>/@title	"abcGroup"	new (attribute) value	<x> start title="abcGroup" <a/> <b/> <c/> end </x>

The **Operation **in the first row is applied to the simple, abstract XML snippet:

<x> </x>

The **Operation **in each succeeding row applies to the **Result **of the row immediately preceding it.

### 3.2 An example

XBabelPhish has been vetted with use cases in the MAGE-ML document domain drawn from: the Stanford Microarray Database [9], the Computational Biology and Informatics Laboratory (CBIL) at the University of Pennsylvania [10], and European Bioinformatics Institute (EBI) Microarray Informatics Group, which runs the ArrayExpress microarray database [11]. Using its split/merge capability, it has translated fairly large (~250 Mb.) documents in translations that apply tens of thousands of changes. For other documents, it has demonstrated the effective use of complex FLWOR expressions in XQuery. Following is a modest example.

#### 3.2.1 Translation definition

**Figure 3:**
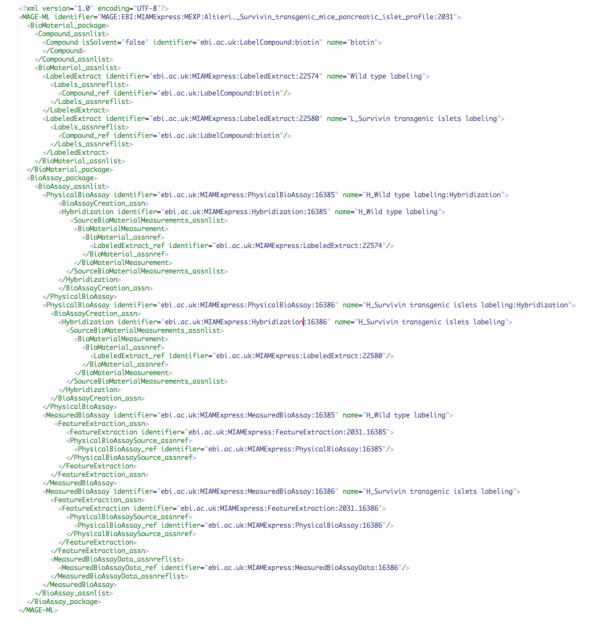
mage-ml_example.xml. Snippet of a MAGE-ML document before XBabelPhish executes the translation trans_example.xml in Figure 1. This valid MAGE-ML contains two *FeatureExtraction *elements (under the *MeasuredBioAssay *elements) that have an *identifier *attribute but no *name *attribute. The translation adds a name attribute to each of them.

Figure 1 presents an illustrative one-step translation definition. The comment at the top of the definition explains the transformation that this step implements. This translation definition should be examined together with the source XML document, presented in Figure 3.

The single step in this translation defines:

1) The set of locations where changes may be made with a **changeLocationQuery **– in this case, a simple XPath expression that selects all *FeatureExtraction *elements within *MeasuredBioAssay *elements.

2) The new content – the value of a new name attribute for each of the *FeatureExtraction *elements selected by the **changeLocationQuery **– with a **locationValueQuery**. In this case, the **locationValueQuery **is a FLWOR expression with three bound variables that constructs the value of a new name attribute by concatenating the values of two other, existing attributes (a *Hybridization *name and a *Compound *name) connected by a string literal. XBabelPhish executes this XQuery once in the context of each selected *FeatureExtraction *element. As a result, the value of the new name for each *FeatureExtraction *is "customized" for that particular element.

3) The operation (**op**) that uses the new content – in this case, **addAttribute**.

4) The name of the new attribute (**newNodeName**) – in this case, "name".

#### 3.2.2 Source and target

Figure 3 is a snipped, but valid XML (and valid MAGE-ML) source document. Only areas relevant to the translation are shown.

**Figure 4:**
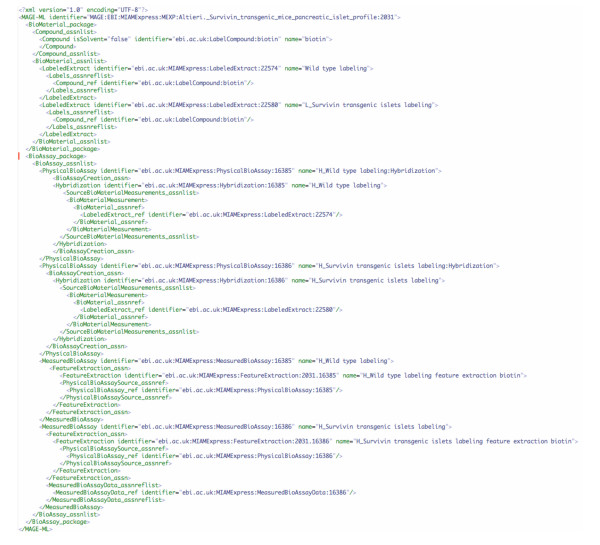
new_example.xml. The result of executing the translation defined by trans_example.xml (Figure 1) on mage-ml_example.xml (Figure 3). The value of a new *name *attribute for each of the two *FeatureExtraction *elements has been computed from their related *Hybridization *and *Compound *elements according to the translation definition.

Figure 4 is XBabelPhish's translation of the source document (Figure 3) according to the translation definition (Figure 1). A comparison of the translated document (Figure 4) with the original (Figure 3) shows that it differs by its addition of a name attribute to each of two *FeatureExtraction *elements. As specified by the translation, each of the new FeatureExtraction names is composed from the names of elements specifically related to that particular FeatureExtraction.

#### 3.2.3 Run output

**Figure 5:**
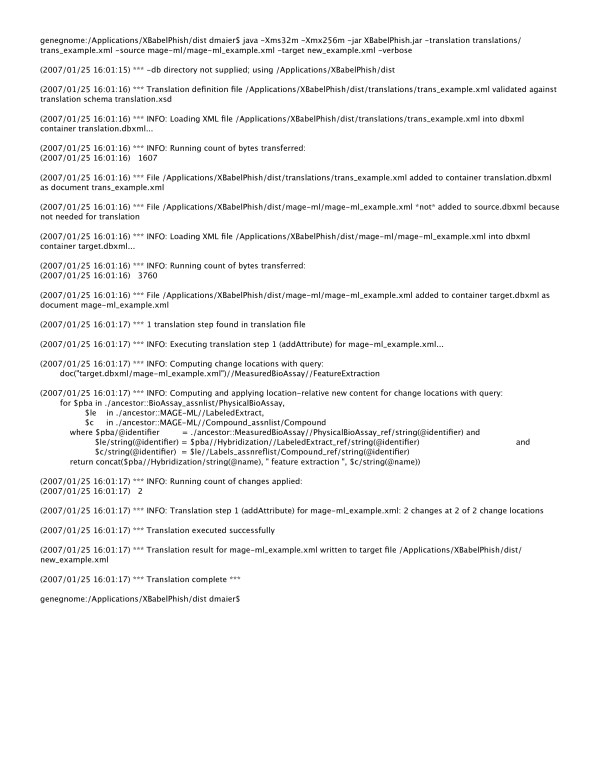
XBabelPhish run output. The running commentary that XBabelPhish gives of its progress executing the translation of Figure 1 on the file snipped into Figure 3. The -verbose option has been used to provide more detail.

Figure 5 is the run output of XBabelPhish performing the example translation. By default, XBabelPhish gives a modest running commentary on its progress. The -verbose option used in Figure 5, provides more details.

## 4 Discussion

### 4.1 Assumptions

Like any other technology solution, the XBabelPhish's design was based on several assumptions concerning the characteristics of a document domain's semantic variants, and the possibility of knowing their differences:

**Limited differences: **There are a manageably small number of differences between the rule sets used by the constructors for distinct semantic variants – typically fewer than 20.

**Limited number of semantic variants: **There are a manageably small number of semantic variants. This is important because this determines the number of translations that a document domain needs – either n (n - 1) pairwise translations or 2n canonical translations for n semantic variants.

**Difference discovery: **It is possible and practical for domain experts to discover differences in the rules that two different producers (see Section 1) use – where rules in the corresponding producers map the same piece of domain (e.g. microarray) information into different XML (e.g. MAGE-ML) elements, attributes or text.

It is possible, and in fact may be common to discover *differences *between two sets of construction rules without necessarily being able to characterize either set of rules independently.

**Using difference information: **It is possible and practical for domain experts to clearly *characterize *the differences between a translation pair of semantic variants in terms of their XML schema [12] or DTD [13], and to *convey *these differences to computer experts. (Note that a DTD is easily converted to a schema using a tool such as trang [14].) The ability to *characterize *these differences hinges on how simple or complex the differences are, and how simply they can be couched in terms of XBabelPhish's primitive operations. The ability to *convey *the differences would likely (though not necessarily) be present when these experts were one and the same person.

### 4.2 Experience

Experience with XBabelPhish in the MAGE-ML document domain is still limited. However, domain experts have so far shown a good ability to understand how various semantic variants differ from their preferred variant. To date, no domain expert has also tried to assume the role of translation definer.

The process of conveying domain knowledge of semantic differences to someone able to formulate the XQuery statements in an XBabelPhish translation definition has been slower and more interactive than anticipated. But, as can be seen from the example, this has been done for nontrivial translations. Since this example and other more complex ones have been drawn from real MAGE-ML translation use cases, this is good evidence that XBabelPhish can address the real translation needs for MAGE-ML documents.

### 4.3 Other approaches

XBabelPhish uses a native XML database for storing XML documents in conjunction with XQuery, with update extensions, for transforming stored documents. This is not the only approach for inter-translating XML semantic variants. A number of alternative approaches were considered, and put aside for various reasons. Approaches vary in their use of different storage vehicles and transformation languages, or in their use of different XQuery engines. The following survey is a small sample:

#### 4.3.1 XSLT

The tool most obviously comparable to XBabelPhish is XSLT [3] – a W3C language for transforming XML documents. Unlike XBabelPhish, XSLT is not intended to be a general-purpose tool. Rather, it is designed for the kinds of transformations most commonly needed for XSL [15] style sheets. In line with this limited goal, XSLT relies on XPath [4], which is a small proper subset of the more powerful XQuery language that XBabelPhish uses. Just as telling, existing XSLT implementations cannot handle documents anywhere near the size commonly encountered in XBabelPhish's initial document domain of MAGE-ML.

#### 4.3.2 LDAP directories

One approach is to store MAGE-ML documents in LDAP directories, translate them within this storage vehicle, and then extract them. There are open source LDAP implementations, such as OpenLDAP [16], and tools that wrap LDAP structures in XML, such as LDAPXML [17]. But a general translation algorithm for XML -> LDAP is problematic. Additionally, the verbs for LDAP transformations are far more primitive that those available through an approach using XQuery-based update.

#### 4.3.3 (Temporary) relational database storage

Another approach is to "shred" MAGE-ML documents into a set of relational database tables, transform the tabularized representation inside the SQL database, and then extract this transformed representation as the translated XML document. There are many variants of this approach, all with major problems. One approach is to use a generalized XML shredding tool that:

1) Generates a relationally efficient SQL schema that preserves both the structural and constraining properties of an XML schema.

2) "Shreds" an XML document that is an instance of the XML schema into a database defined with the generated relational schema.

3) Transforms the shredded document via SQL statements that are automatically generated from XQuery statements based on the XML schema.

4) "Unshreds" the document back into XML.

This is a very difficult problem. To date, there has been no successful implementation of this approach. The most promising effort at the time of our initial investigation was ShreX [18], whose documentation seems to promise much of items 1) – 4). Unfortunately, the only known extant implementation does not fulfill this promise.

#### 4.3.4 Other XQuery engines

Berkeley DB XML is not the only open source XML database/XQuery engine. eXist [19] is another. Unfortunately, when eXist was evaluated, its indexing scheme limited its ability to handle XML nodes with a branching factor greater than the capacity for a 16-bit integer. Large MAGE-ML documents far exceed this limit, and therefore eXist's ability to accommodate them. This limitation was in the process of being fixed (and the fix is already available in a development version of the product). However, some of eXist's supporting tools, for example its Java console-based browser, were also severely limited in capacity.

Yet another XQuery engine is Galax [20]. Unlike Berkeley DB XML and eXist, Galax is *not *coupled with a database. But it includes an implementation of XQuery! [21] for updating XML documents. Its technology probably makes it more capable than most XQuery engines in running queries on extremely large documents. Despite this, simple tests (loading and running simple XPath queries) showed that eXist's performance for larger MAGE-ML documents is marginal. It should be noted, too, that Galax's installation is extremely cumbersome.

## 5 Conclusion

XBabelPhish is a very powerful and efficient tool for inter-translating semantic variants of MAGE-ML, thereby improving the interchange of microarray experiment information. Initial use cases show a promising potential in this document domain. In addition, XBabelPhish is completely independent of MAGE-ML semantics, or indeed the semantics of any document domain. It is perhaps unique in this independence, in its expressive power, and in its ability to translate very large documents.

## Availability and requirements

**Project name: **XBabelPhish XML Translator

**Project home page: **XBabelPhish distribution archive: 

**Operating system(s): **All platforms that Berkeley DB XL supports, including Windows, Linux, BSD UNIX, Mac OS/X and all POSIX-compliant operating systems; not tested on the 64-bit versions of these platforms

**Programming language: **Java (XBabelPhish), C, and C++ (Berkeley DB XML)

**Other requirements: **J2SE 1.5 or newer (implying a need for Mac OS/X 10.4 or newer for Mac users) Berkeley DB XML Version 2.3.8 or newer (2.3.10 recommended as of this writing) Web browser or html viewer for viewing the javadoc

**Open Source Licenses: **XBabelPhish: MIT 

StAX: Apache License 2.0 

Berkeley DB XML: Oracle/Apache 1.1 

Berkeley DB: Oracle/University of California/Harvard for Berkeley 

Xerces: Apache License 2.0 

Pathan: DecisionSoft/BSD 

**Any restrictions to use by non-academics: **Commercial license for Berkeley DB XML needed (available from Oracle).

## Authors' contributions

CAB and GS provided information about the needs of the MAGE-ML-using community. DM determined the specific requirements for XBabelPhish, designed it, implemented it, tested it, wrote all the documentation, and when necessary, helped debug problems in Berkeley DB XML. FW provided some initial use cases and technical consultation. CAB and FW consulted on general approaches to some thorny problems encountered along the way. DM wrote the manuscript, and CAB and GS edited the manuscript and provided comments and suggestions. All authors have read the manuscript and approve of its content.
